# Trial by trial EEG based BCI for distress versus non distress classification in individuals with ASD

**DOI:** 10.1038/s41598-021-85362-8

**Published:** 2021-03-16

**Authors:** Safaa Eldeeb, Busra T. Susam, Murat Akcakaya, Caitlin M. Conner, Susan W. White, Carla A. Mazefsky

**Affiliations:** 1 Swanson School of Engineering, University of Pittsburgh, Pittsburgh, PA USA; 2 School of Medicine, University of Pittsburgh, Pittsburgh, PA USA; 3 University of Alabama, Tuscaloosa, AL USA

**Keywords:** Electrical and electronic engineering, Biomedical engineering

## Abstract

Autism spectrum disorder (ASD) is a neurodevelopmental disorder that is often accompanied by impaired emotion regulation (ER). There has been increasing emphasis on developing evidence-based approaches to improve ER in ASD. Electroencephalography (EEG) has shown success in reducing ASD symptoms when used in neurofeedback-based interventions. Also, certain EEG components are associated with ER. Our overarching goal is to develop a technology that will use EEG to monitor real-time changes in ER and perform intervention based on these changes. As a first step, an EEG-based brain computer interface that is based on an Affective Posner task was developed to identify patterns associated with ER on a single trial basis, and EEG data collected from 21 individuals with ASD. Accordingly, our aim in this study is to investigate EEG features that could differentiate between distress and non-distress conditions. Specifically, we investigate if the EEG time-locked to the visual feedback presentation could be used to classify between WIN (non-distress) and LOSE (distress) conditions in a game with deception. Results showed that the extracted EEG features could differentiate between WIN and LOSE conditions (average accuracy of 81%), LOSE and rest-EEG conditions (average accuracy 94.8%), and WIN and rest-EEG conditions (average accuracy 94.9%).

## Introduction

Autism spectrum disorder (ASD) is a neurodevelopmental condition characterized by impaired social interaction and communication, restricted interests, and repetitive behaviors^3^. Accumulating evidence has indicated differences in processing and connectivity across brain regions in individuals with ASD, relative to non-autistic peers^6,7^. These pervasive impairments have a significant impact on emotional functioning and emotion regulation (ER), which is defined as the person's ability to modify their arousal and emotional state to promote adaptive behavior^9^. Compared to typically developing individuals, individuals with ASD have more impaired ER, which may cause them to react maladaptively to emotional stimuli with tantrums, temper outbursts, aggression and self-injurious behavior^11^. ER impairment contributes to negative social outcomes, which in turn leads to negative expectancies for future interactions and a cycle of social avoidance, missed opportunities, and failure to learn new social skills as well as, ultimately, poor quality of life marked by limited independence^12–17^. Given the pervasive impact of impaired ER across functional domains, it should be considered while developing ASD treatment protocols^19^.

There has been an increasing interest in using neurofeedback technology in the treatment and study of several neurodevelopmental conditions such as ASD. Electroencephalography (EEG) based neurofeedback can provide an assessment and intervention tool that is safe, portable, and easy to use without the need for verbal or physical response^22,23^. It also provides high temporal resolution, allowing real time experience to its users while interacting with virtual social environment that involves emotion expression, recognition and regulation. Furthermore, it can be used to engage children with ASD in life-like social situations through augmented reality, in the safety of one’s own home. Augmented neurofeedback can also be used to provide augmented traditional therapy sessions similar to the in-person sessions, which would reduce the cost and increase the accessibility to more children^22–24^.

Various studies have assessed the effectiveness of using EEG in neurofeedback-based interventions for individuals with ASD^25–27^. EEG-based neurofeedback has been successfully used to improve cognitive flexibility, and enhanced social and communicative skills in individuals with ASD^25–27,29,30^. EEG-guided neurofeedback studies demonstrated changes in the EEG activity in individuals with ASD compared to neurotypical individuals^1,2,4^. Murias et al.^31^, found an increase in the theta and beta band powers in individuals with ASD’s EEG activity compared to their neurotypical peers during resting state. Another study by Kouijzer et al.^29^ showed a significant improvement in social and communication skills when the power in the theta band in individuals with ASD was reduced. Cowan et al.^30^ found a substantial improvement in attention and socialization after suppressing the excessive alpha and theta power. Furthermore, Wang et al.^27^ showed a linear decrease of the theta to beta power ratio, and an increase in the gamma power after applying the neurofeedback intervention.

Several EEG components have been identified which are associated with emotional processing and regulation in individuals with ASD and neurotypical individuals^1,2,4,5,8,10,18,20,21,28^; a summary of these EEG features is shown in Table 1. EEG components such as the frontal theta oscillations and power were shown to be associated with ER for both neurotypical individuals and individuals with ASD^1,5^. It has been found that ER induced by negative pictures showed an increase in frontal theta oscillations^5^. Moreover, a positive correlation between theta oscillations and successful usage of cognitive reappraisal to decrease negative emotions has been shown^26^. Another study by Noordt et al.^1^ showed that inter-trial coherence of medial frontal theta oscillations was significantly lower for individuals with ASD. They also showed that the medial frontal theta power and phase coherence were greater following LOSE compared to WIN feedback. Moreover, they found that individuals with ASD were more sensitive to the valence of the reward as compared to their neurotypical peers. Another study by Stavropoulos et al.^2^, investigated the power in alpha and theta bands in response to a reward prediction task in both individuals with ASD and neurotypical individuals. They observed suppression in the alpha band power after presenting the feedback in individuals with ASD, while more theta activity was observed in neurotypical individuals.

**Table 1:** Studies of EEG patterns associated with emotional processing and regulation.

Author	Studied population	EEG feature	Stimuli type/feedback	Findings
Noordt et al.^1^	ASD group (N = 27) NT Group (N = 22)	Event-related spectral perturbations (ERSPs) Inter-trial coherence of medial frontal theta oscillations	Feedback-Reward Paradigm	Lower inter-trial coherence of medial frontal theta for ASD group Medial frontal theta power and phase coherence were greater in LOSE feedback
Stavropoulos et al.^2^	ASD group (N = 20) NT group (N = 23)	Alpha and Theta band power	Feedback-Reward Paradigm	Greater alpha suppression in ASD compared to NT after feedback More theta band activity during reward processing in NT group
Larson et al.^4^	ASD group (N = 25) NT group (N = 25)	Amplitude of FRN	Monetary loss/gain feedback	Larger FRN in response to loss trials than gain trails in both groups
Ertl et al.^5^	NT group (N = 30)	Frontal theta oscillations	Negative and neutral images	Increase in frontal theta oscillations in prefrontal brain regions
Thiruchselvam et al.^8^	NT group (N = 18)	LPP	Negative images	Reappraisal and Distraction reduced the amplitude of LPP
Cuthbert et al.^10^	NT group (N = 37)	LPP P300	Emotional images (pleasant and unpleasant) and neutral images	Emotional stimuli increased the amplitude of LPP Pleasant emotional stimuli increased the amplitude of P300
Lerner et al.^18^	ASD group (N = 34)	Latency and Amplitude of N170, N250, N100 N300, P200	Facial emotion stimuli and vocal emotion stimuli	Positive significant correlation with N170 latency, and facial stimuli Significant correlation with N100 latency and voice stimuli
Whitehouse et al.^20^	ASD group (N = 15) NT group (N = 15)	Latency and Amplitude of P100, N200, P300 N400	Speech sound stimuli Nonspeech sound stimuli	Reduced amplitude P100, N200, P300 N400 during perception of speech sound in children with ASD
Dawson et al.^21^	ASD group (N = 29) NT group (N = 22)	Amplitude and latency of N300, P300, P500	Neutral and fear expression images	Larger N300 response to a fear face than neutral face in NT compared to ASD group
Hileman et al.^28^	ASD group (N = 20) NT group (N = 20)	Amplitude and latency of P1 and N170	Facial expression and vehicle images	Larger P1 and N170, shorter P1 and N170 latency in NT compared to ASD

ASD group: Individuals with ASD, NT group: Neurotypical individuals. N: the number of participants.

Other studies of changes in the event related potentials (ERPs) have shown that emotional stimuli induce P300^10^ and late positive potential (LPP)^8,32^ in neurotypical individuals. P300 is attention dependent, and therefore reflects higher cognitive processing of stimuli^32,33^, while LPP is more sustained positivity. Thiruchselvam et al.^8^ studied the effects of negative response inhibition and reappraisal on amplitude and latency of P300 and LPP in neurotypical individuals. Findings of this study showed a decrease in the amplitude of P300 and the duration and amplitude of LPP. Another ERP component is the feedback-related negativity (FRN), which reflects the neural activity associated with task performance and outcome monitoring^34^. Larson et al^4^. demonstrated that the amplitude of the FRN in response to distress conditions is different from the non-distress conditions both for individuals with ASD and neurotypical individuals during the monetary loss- gain feedback^4^. Therefore, FRN amplitude could be utilized to identify conditions associated with distress^35^.Overall, it is well established that earlier responses and larger amplitudes of ERP reflect more intact functioning in both ASD and neurotypical individuals^28,36,37^. Hileman et al.^28^ examined the difference in the P1and N170 in response to emotion stimuli in children with ASD and their neurotypical peers. Findings indicated that neurotypical individuals showed a larger amplitude and shorter latency of P1 and N170 in response to emotion stimuli compared to their peers with ASD. These studies show that several EEG components could be used to detect and monitor changes in brain activity related to ER in neurotypical and individuals with ASD.

In this study, we developed an EEG-based BCI system based on an Affective Posner^38,39^ task to analyze the effect of distress on the brain activity of individuals with ASD. Identifying brain responses to distress is a foundational step towards developing BCI technology tools to support ER-focused interventions. The Affective Posner task with deception has been used previously in functional magnetic resonance imaging studies for ER through the participation of chronically irritable children^44^, but not in EEG-based studies. Our preliminary results showed that the extracted set of temporal and spectral EEG features showed high accuracies in differentiating between distress (LOSE) and non-distress (WIN) conditions (average accuracy of 81%), and the LOSE and rest-EEG conditions (average accuracy 94.8%), and WIN and rest-EEG conditions (94.9%), with low variance across participants.

## Results

### Features analysis

The weighted sequential forward feature selection (WSFS) algorithm selects a subset of features that significantly contribute to the accurate classification of each problem. In this work, we have three main classification problems as shown in Fig. 3. Representing distress and non-distress as LOSE and WIN conditions respectively, we investigate the possibility of extracting EEG features that could distinguish between: (1) LOSE and rest-EEG (baseline) conditions; and, (2) WIN and rest-EEG (baseline) conditions; (3) LOSE and WIN conditions.

**Figure 1:**
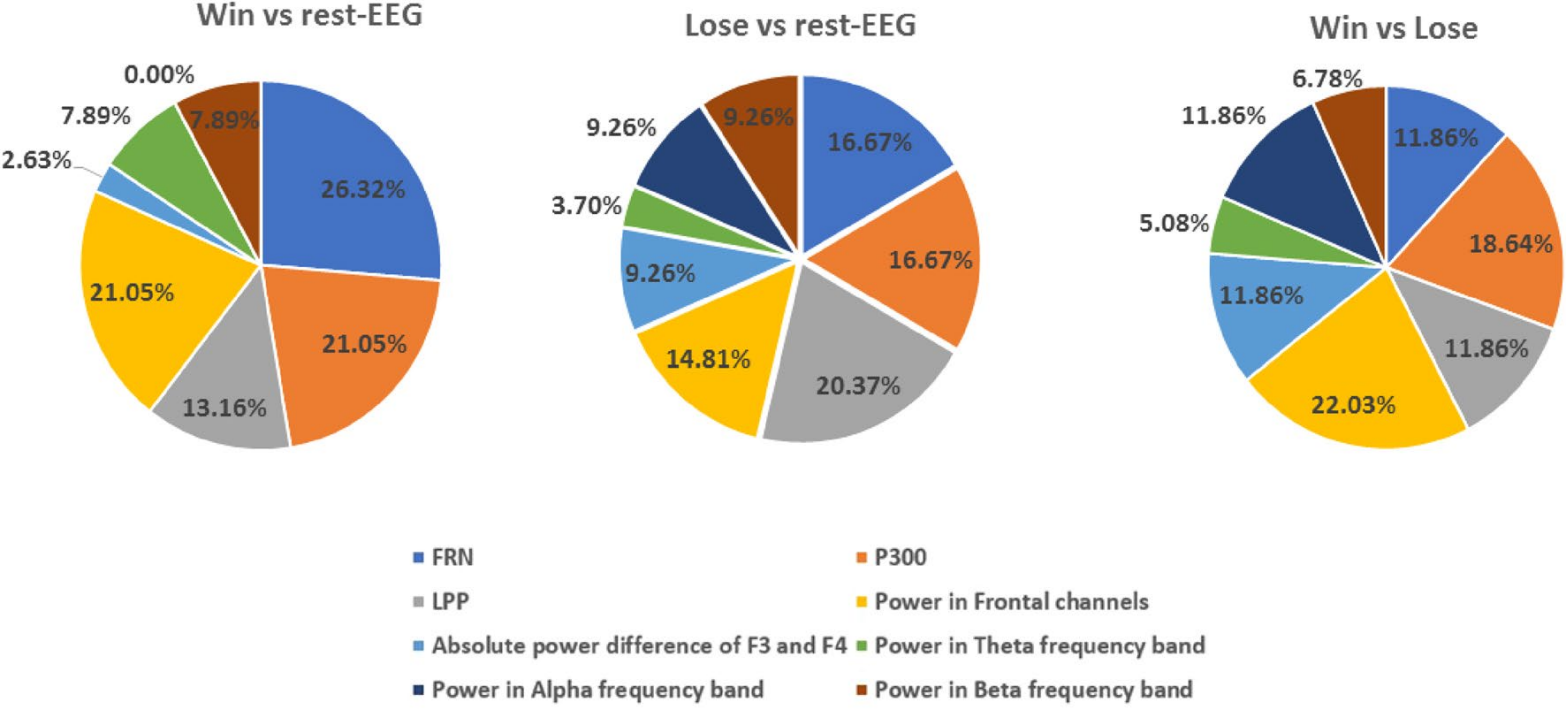
The percentage of the selected features for each classification problem.

The percentage of the occurrence of feature subsets of each classification problem generated by applying the WSFS algorithm is shown in Fig. 1. The generation of this pie chart is discussed in the methods section. For the first classification problem, WIN versus LOSE conditions, both the power in frontal channels in the frequency range (4–30 Hz) and the P300 EEG features showed the highest percentage of selections by the WSFS algorithm. The power in frontal channels showed high contribution towards the first classification problem with 22.03%, and the P300 showed high significance with 18.64%. Moreover, The FRN, LPP and power in alpha frequency band showed high significance during the classification of distress and non-distress conditions with 11.86%.

For the classification problem of differentiating between the LOSE and rest-EEG conditions, the LPP, the P300 and total power in the frontal channels showed high contribution towards the overall accuracy, with 20.37%, 16.67% 14.87% respectively. Moreover, the WSFS algorithm showed that the P300, FRN and the total power in the frontal channels in the EEG frequency range (4–30 Hz) contribute significantly towards the classification of WIN versus rest-EEG conditions. The late positive potential, P300 followed by the FRN and the total power in the frontal channels in the EEG frequency range (4–30 Hz) are the EEG features most contributed in classifying between LOSE and rest-EEG conditions.

### Analysis of lose and rest EEG conditions

The performance measurement of LOSE versus rest-EEG (baseline-EEG) classification, for each participant are presented in Table 2. The performance metrics include the accuracy, sensitivity and F1 scores^40^. These results were calculated using the subset of features that resulted in the highest classification rate after cross validation for each participant. The overall average classification accuracy is 94.79% (chance level of 50%), while the average sensitivity of LOSE is 92.6% with low variance as shown in Table 2. The average sensitivity of the rest-EEG conditions is 94.55% across all participants. F1-scores shows an overall average value of 0.9 across all participants, with a minimum value of 0.69 and maximum value of 1.

**Table 2:** The accuracy and sensitivity of LOSE and rest-EEG classes, and F1 score results for each participant for task 3.

ID	Accuracy	Sensitivity (lose)	Sensitivity (rest-EEG)	F1-score
1	99.41	100	99.16	0.99
2	93.16	90.77	94.42	0.89
3	98.92	96.92	100	0.98
4	92.86	92.73	93.33	0.95
5	97.5	100	86.66	0.98
6	82.93	81.25	84.00	0.78
7	96.02	96.36	95.00	0.97
8	87.51	96.00	73.33	0.90
9	95.38	96.00	93.33	0.96
10	98.82	98.46	100	0.99
11	88.75	90.77	80.00	0.92
12	96.31	89.23	100	0.94
13	100	100	100	1
14	99.31	96.00	100	0.97
15	100	100	100	1
16	100	100	100	1
17	89.23	75.71	96.82	0.56
18	98.38	95.00	100	1
19	96.31	90.77	99.23	0.87
20	93.71	88.00	96.00	0.73
21	86.31	70.77	94.42	0.69
${\text{Mean}} \pm {\text{SD}}$	94.79 ± 4.99	92.6 ± 7.9	94.55 ± 7.3	0.9 ± 0.11

### Analysis of win and rest EEG conditions

The accuracy, sensitivity and F1 scores for the 2-class classification, WIN versus rest-EEG (baseline-EEG) conditions, for each participant are presented in Table 3. Similar to the previous classification problems, these results were calculated using the sub-set of features that resulted in the highest classification rate after cross validation for each participant. The overall average classification accuracy is 94.9%, while the average sensitivity of WIN condition is 90.5% with low variance as shown in Table 3. The average sensitivity of the rest-EEG conditions is 93.1% across all participants. F1-scores shows an overall average value of 0.9 across all participants, with a minimum value of 0.68 and maximum value of 1.

**Table 3:** The accuracy and sensitivity of Win and rest-EEG classes, and F1 score results for each participant for task 3.

ID	Accuracy	Sensitivity (Win)	Sensitivity (rest-EEG)	F1-Score
1	100	100	100	1
2	98.00	93.31	99.23	0.94
3	96.23	91.43	97.22	0.90
4	82.52	100	53.31	0.87
5	100	100	100	1
6	88.31	68.92	95.22	0.75
7	100	100	100	1
8	86.43	100	73.31	0.89
9	95	92.00	100	0.95
10	98.21	97.00	100	0.98
11	82.22	100	46.72	0.88
12	98.74	94.32	100	0.96
13	100	100	100	1
14	100	100	100	1
15	100	100	100	1
16	100	100	100	1
17	88.42	43.43	99.25	0.57
18	100	100	100	1
19	93.73	94.32	93.62	0.87
20	95.00	73.42	97.61	0.73
21	90.92	53.41	100	0.68
${\text{Mean}} \pm {\text{SD}}$	94.9 ± 5.6	90.5 ± 16	93.1 ± 15	0.9 ± 0.12

### Distress and non-distress conditions analysis

The performance measurement of distress (LOSE) versus non-distress (WIN) condition classification for each participant are presented in Table 4. These results were calculated using the sub-set of features that resulted in the highest classification rate after cross validation for each participant. The overall average classification accuracy is 81.36% (chance level of 50%), while the average sensitivity of distress condition is 89.97% (with low variance as shown in Table 4). The sensitivity of the non-distress conditions is lower than that of the distress conditions with average sensitivity value of 63.4% for all participants. F1-scores shows an overall average value of 0.68 across all participants, with a minimum value of 0.51 and maximum value of 0.92.

**Table 4:** The accuracy and sensitivity of distress (LOSE) and non-distress (WIN) classes, and F1 score values for each participant results for task 3.

ID	Accuracy	Sensitivity (LOSE)	Sensitivity (WIN)	F1 score
1	65.88	68.00	62.86	0.60
2	81.05	92.31	56.66	0.65
3	74.00	78.46	65.71	0.65
4	80.00	96.37	44.00	0.56
5	74.00	87.73	48.62	0.56
6	76.82	88.75	55.55	0.61
7	70.58	85.45	43.33	0.51
8	92.53	92.00	93.33	0.90
9	82.66	84.00	80.00	0.75
10	75.00	92.31	42.86	0.53
11	87.37	90.77	80.00	0.80
12	80.00	87.67	65.71	0.70
13	78.95	90.77	53.33	0.61
14	95.00	100	86.66	0.92
15	77.92	80.00	74.32	0.71
16	81.00	95.72	46.66	0.6
17	88.00	91.43	80.00	0.80
18	87.52	100	50.00	0.66
19	84.00	92.31	68.58	0.75
20	92.31	100	66.66	0.78
21	86.31	95.38	66.66	0.74
${\text{Mean}} \pm {\text{SD}}$	81.46 ± 7.32	89.97 ± 7.59	63.4 ± 14.68	0.68 ± 0.11

### Validation results

The results of applying Wilcoxon rank statistical test on the feature vectors show that the total frontal power and absolute power difference of F3 and F4 (*p* value of 8.74e−04 and 0.0234) were significant for Win versus lose classification while P300 was not significant (*p* value of 0.5223). For Lose versus rest-EEG classification, total frontal power and LPP were significant features (*p* value of 3.7483e−200 and 7.1587e−14); however, P300 did not show any significance (*p* value of 0.947). In the classification of Win versus rest-EEG, only total frontal power was significant (*p* value of 8.7714e−101). The results for the Wilcoxon rank sum test performed over SVM scores of Win versus Lose, win versus rest-EEG and Lose versus rest-EEG classification showed that SVM scores were significant in each classification problem (*p* values of 1.3287e−12, 0 and 1.9545e−179). Note that, zero is the observed *p* value in MATLAB when the *p* value is smaller than the smallest number that could be presented in MATLAB.

In order to validate the significance of the features that are selected through WSFS (feature selection algorithm) on the classification performance, we applied a permutation test within each classification problem. In this permutation test, we randomly assign features to each participant for each classification problem, and this assignment is repeated 100 times. The results of the permutation test are shown in Table 5. Average (average over 100 repetitions) performance measures (accuracy and F1 score) were calculated for Win versus Lose, Lose versus rest-EEG and Win versus rest-EEG classifications for each participant. Moreover, the results of the right-sided Wilcoxon rank test performed after the permutation task as discussed in the validation subsection of the methods section (comparing Table 5 results with results of Tables 2, 3 and 4), show that the accuracies obtained through WSFS algorithm are significantly higher than the average accuracy obtained through random feature assignment with p value of 1.87E−07, 5.51E−08, 1.74E−05 in Win versus Lose, Lose versus rest-EEG, and Win versus rest-EEG classification, respectively. Similarly, F1 score obtained through WSFS algorithm significantly differ than the average F1 score selected by random feature assignment by p value of 1.16E−08, 5.30E−06, 4.44E−06 in Win versus Lose, Lose versus rest-EEG, and Win versus rest-EEG classification, respectively.

**Table 5:** The accuracy and F1 score for each classification problem obtained through permutation and random feature assignment for each participant.

ID	Win versus lose		Lose versus rest-EEG		Win versus rest-EEG	
	Accuracy	F1 score	Accuracy	F1 Score	Accuracy	F1 score
1	57.44	0.11	74.33	0.18	83.77	0.32
2	68.23	0.00	71.38	0.30	81.42	0.06
3	64.19	0.01	85.52	0.78	83.16	0.33
4	68.03	0.00	84.37	0.90	70.45	0.78
5	64.00	0.00	79.79	0.89	72.01	0.82
6	65.37	0.02	64.53	0.28	74.66	0.00
7	66.42	0.00	77.13	0.87	64.19	0.76
8	60.39	0.00	62.82	0.74	56.50	0.19
9	66.26	0.00	77.42	0.87	64.59	0.75
10	65.26	0.00	86.83	0.92	84.85	0.89
11	67.04	0.03	80.31	0.89	67.16	0.78
12	62.90	0.00	81.38	0.58	84.49	0.41
13	65.79	0.00	80.62	0.67	95.04	0.78
14	62.58	0.02	92.80	0.69	97.95	0.78
15	63.67	0.02	80.31	0.56	95.35	0.81
16	70.61	0.00	74.67	0.38	94.44	0.73
17	69.02	0.00	65.50	0.17	80.36	0.00
18	73.76	0.00	77.31	0.50	86.66	0.11
19	64.94	0.01	82.78	0.73	89.07	0.69
20	78.79	0.00	75.98	0.43	90.26	0.03
21	68.89	0.00	69.31	0.25	81.30	0.03
Mean ± SD	66.36 ± 4.53	0.11 ± 0.02	77.39 ± 7.61	0.60 ± 0.26	80.84 ± 11.55	0.48 ± 0.34

## Discussion

The research objective of this study is to identify and analyze EEG features that are able to differentiate between the distress and non-distress conditions of individuals with ASD. For this purpose, the study protocol involved a card game with deception. We analyzed and extracted temporal and spectral EEG features from the collected EEG time-locked to the feedback presentation. Our approach includes a systematic feature selection, the WSFS algorithm, that selects a subset of EEG features that contributes towards the overall accuracy of each classification problem. Therefore, an initial set of temporal and spectral features were selected based on previous studies of cortical activity related to ER of neurotypical individuals and individuals with ASD^1,2,4,5,8,10,18,20,21,28^. This set of features includes spectral features as follows, the total power in alpha, theta, beta frequency bands and in the frequency range (4–30 Hz). Moreover, a number of temporal features have been also calculated, the P300, the LPP and the FRN. Then, a subset of these features was chosen based on their ability to provide information about each classification problem. More specifically, we analyzed the EEG recorded during the task where feedback was predetermined to result in LOSE feedback (too slow, wrong) or WIN (correct). The WSFS algorithm started with a single feature and added other features satisfying high overall accuracy, as explained in Fig. 4.

The findings of this study suggest that both the power in frontal channels in the frequency range (4–30 Hz) and the P300 EEG features are very informative and contribute significantly towards the classification of distress and non-distress conditions for individuals with ASD. The P300 showed the highest contribution towards the classification of rest-EEG versus LOSE conditions, and rest-EEG versus WIN conditions. These findings align with previous studies in neurotypical populations^8,32,41,42^, where it was shown that positive emotional stimuli introduces P300 and LPP, and the negative response inhibition and reappraisal decreases the amplitude of P300 and affect the duration and amplitude of LPP. Therefore, we argue that P300 and LPP are both ERPs of EEG that could be utilized as a measure of ER^8,32,41–43^.

On the other hand, we found that in addition to the P300 and total frontal power, the FRN showed a high significance in classifying the WIN versus rest-EEG and LOSE versus rest-EEG conditions. These ERPs are sensitive to visual stimuli and they have been used for stimulus identification^44^ and visual stimuli classification^45^. Moreover, the absolute power difference between the EEG channels F3 and F4 showed high sensitivity towards the classification of LOSE conditions in both WIN versus LOSE and LOSE versus rest-EEG classification problems. Absolute power difference between the frontal channels^46^ have been used in emotion studies and these studies^47,48^ showed that anxiety and distress levels are reduced by increasing left compared to right prefrontal power. Therefore, in addition to P300 and LPP, absolute power difference between F3 and F4 is also a feature to be further investigated for ER. Moreover, the results of the Wilcoxon rank test applied on the set of features selected by the WSFS algorithm to assess their significance in the classification of Win versus rest-EEG, Lose versus rest-EEG and Win versus Lose showed that only certain features that appeared in the list of top 3 selected EEG features reached statistical significance. However, these selected features go through nonlinear transformation where SVM scores are extracted during classification and they contribute to the classification between conditions after this nonlinear transformation. We argue that such a classification approach and extracting new nonlinear combinations of these selected features to obtain SVM scores is very informative. Therefore, we performed Wilcoxon rank sum test over SVM scores of Win versus Lose, Win versus rest-EEG and Lose versus rest-EEG classification with significance level of 0.05. As a result, SVM scores were significant in each classification procedure. Furthermore, we applied permutation task where the features randomly assign to each participant in each classification problem. It is also important to note that during the random assignments, each participant had a subset of features different from the features selected by WSFS algorithm. The Wilcoxon rank sum test revealed that the features selected though WSFS algorithm were more significant than the features obtained through permutation task for each classification. Overall, these findings confirm that the features selected by WSFS algorithm was significant for each classification problem.

Changes in the above-discussed EEG features provided very high performance (accuracy, sensitivity and F1-scores) of WIN and LOSE condition detection compared to rest-EEG as also was shown in Tables 2 and 3. On the other hand, as shown in Table 4, the average accuracy of classifying between LOSE and WIN conditions is 81.46% (chance level:50%) with high sensitivity of detecting LOSE conditions of 89.9% (with low variance across participants). The sensitivity of the WIN conditions is slightly above chance level, which could be explained as we perform trial-by-trial classification and WIN and LOSE conditions directly follow each other during the game. This in return may affect the separation between the LOSE and WIN conditions. There is evidence of sustained neural processing following the presentation of negatively-valanced stimuli in ASD^49^ so it is plausible that the EEG features reactive to LOSE trials are still reactive during the quick transition to a WIN trial. Further, while receiving “correct” cues during the WIN trial, it is the case that participants still may be below the overall amount required to win their bonus, therefore leading to sustained negative emotion. Taken together, it is not surprising to find lower sensitivity to differentiating WIN versus LOSE and high sensitivity to differentiating both WIN and LOSE from rest-EEG. In summary, the introduced set of features showed a high contribution towards the classification of distress, non-distress and rest-EEG conditions on a single trial basis which suggests that these features can be monitored in real-time through this EEG-based BCI to detect changes in distress for ER intervention.

Our overarching goal is to eventually utilize this EEG-based BCI system as an ER intervention tool to tightly complement psychotherapeutic clinical treatments. While medications are considered a common and effective treatment for ER impairments, the use of medication presents with adverse side effects such as sedation and a significant relapse rate^50^. One promising manualized psychotherapy intervention to improve ER in those with ASD is the Emotion Awareness and Skills Enhancement (EASE) program^51^. EASE targets impaired ER during the high-risk transitional period of adolescence into young adulthood. Evaluation of EASE to date has found that it is feasible to implement, is acceptable to consumers, and results in medium to large effects for the reduction of ER impairments as well as associated depression, anxiety, and problem behaviors^51^ As evidence-based treatments for ER in ASD become available, there is need to consider how to translate these tools into clinical practice and support dissemination of key therapeutic ingredients. As such, there is a growing opportunity and need for complimenting such behavioral clinical treatments with various low-cost and easy-to-access technology-based tools. The proposed EEG-based BCI system is portable, cost effective and it also has very high temporal resolution. Our next steps will be to directly integrate EASE strategies in the EEG-based BCI and provide users with opportunities to repeat the learned clinical skills during simulated training for ER and distress tolerance. The classifier developed and presented in this manuscript will be the foundation of real-time (trial-by-trial) monitoring of potential increase in the distress level of the individuals with ASD, and when an increase in distress level is detected, the envisioned BCI system will operate to provide stimuli/cues (i.e., visual stimuli) to trigger the use of emotion regulation strategies learned through EASE. The overarching goal of this envisioned EEG-based BCI is to improve EASE’s generalization effects to activities outside of the therapy environment for adolescents and adults with ASD. Accordingly, this will be the first EEG-based BCI specifically designed for ER intervention for individuals with ASD, which will hopefully increase accessibility to intervention where specialists may be harder to find, in a format that is likely to appeal to users and that is based on underlying biology.

## Methods

### Experimental procedure

#### Participants

A total of twenty-one participants with ASD were recruited and their guardians provided written informed Consent approved by the University of Pittsburgh Institutional Review Board (IRB #STUDY17070496). Inclusion criteria as follows (1) ages 12–21, inclusive; (2) a clinical diagnosis of ASD, confirmed by research reliable administration of the Autism Diagnostic Observation, Second Edition (ADOS-2)^52^. During the consent process, the participants were informed that their safety and the confidentiality of the collected data are the primary consideration. Participants were told that at any point during the experimental procedure, if they feel any discomfort, they could stop the experiment. Moreover, all the experimental procedures described below is approved by the University of Pittsburgh Institutional Review Board. All participants received $25 for participation, plus an additional of $50 for winning.

#### Affective Posner task

**Figure 2:**
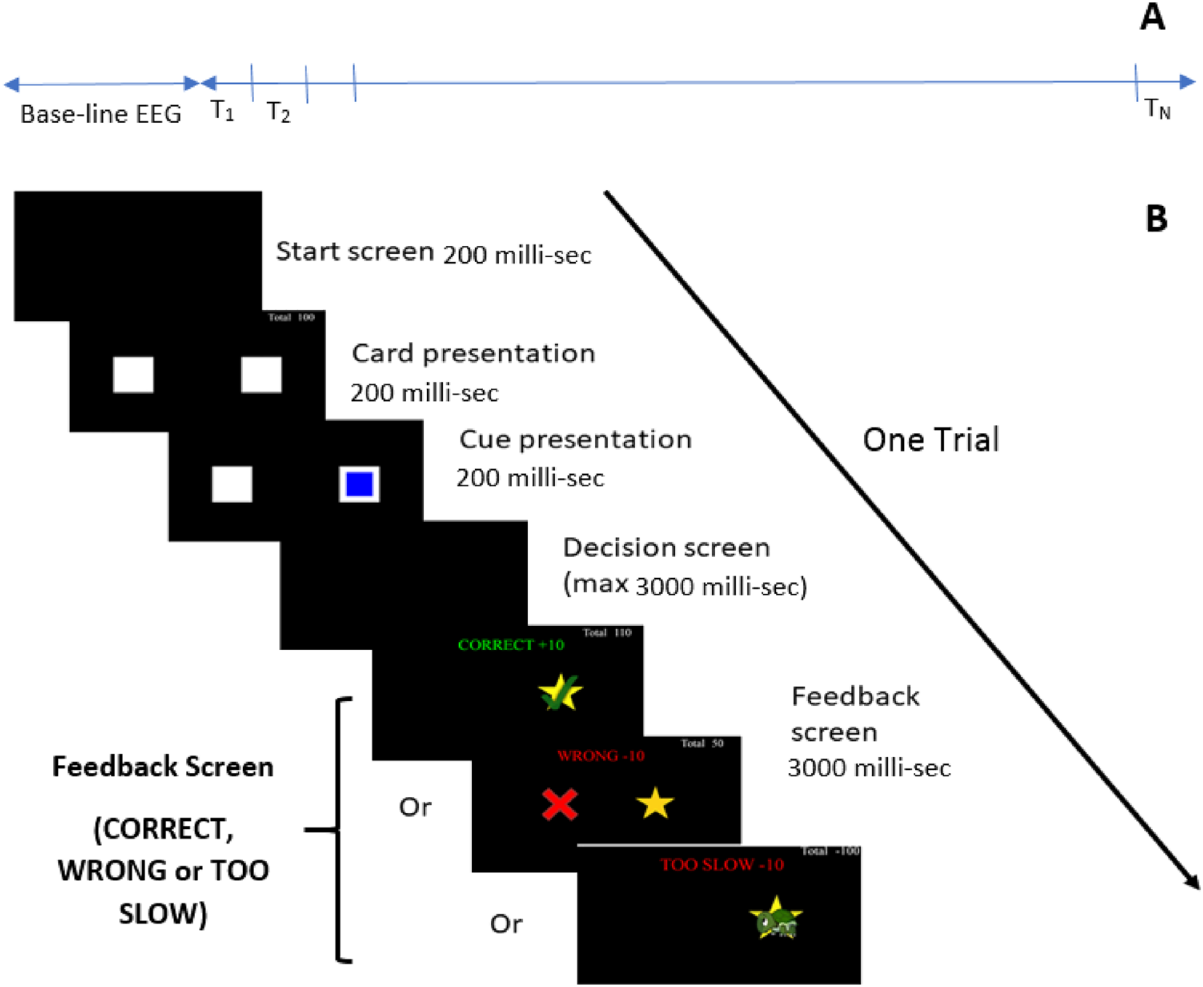
(**A**) Task three and four paradigms, where T refers to a complete trial, (**B**) the proposed complete task-trial based on the Affective Posner with deception. For task one and two, there is no baseline EEG and the feedback of the first task doesn’t include the too slow feedback screen.

Participants were seated in a comfortable chair facing a computer screen and were asked to play a card game based on an Affective Posner task^38,39^ as shown in Fig. 2. The game consists of four tasks, where each task is composed of *N* number of trials. The first two introductory tasks were completed without EEG data collection and without any deception, and each of the first two tasks consisted of 50 trials. The first task was developed to learn the response time of each participant, where only Correct or Wrong feedback were presented. The average response time per each participant was estimated and used in task 2 which included the three feedbacks (Correct, Wrong, Too Slow). In this second task, the Too Slow feedback is presented if the player took longer time to decide the location of the star than their averaged response time learned in task 1. The third task includes deception and EEG data collection. Two-minutes of resting-state EEG was collected as baseline before task 3. Third task has a deception component where 60% of all the correct responses led to “Too Slow” feedback, independent of the actual participant’s response time. To induce an emotional reaction, participants were motivated by a potential bonus of $50 which was dependent on earning a sufficient number of points greater than zero. Participants started with a total of 150 points, and this task is composed of 100 trials. If the participant received a Correct response during the third task, 10 points were added to the total points. If Wrong or Too Slow feedback was presented, 10 points were taken off the total points. If the total number of points was greater than 0, the participant was considered a winner and qualified for the $50 bonus. The third task was designed such that the total number of points was always less than zero at the end of this task. One-minute of resting-state EEG followed task 3, followed by a task set similar to task three but without deception, to ensure that the participant win a sufficient number of trials (points) to earn the $50 bonus. Figure 2A shows the proposed task paradigm, where baseline EEG collection occurs before tasks 3 and 4, followed by a series of trials for each task. Each trial, *T*, consists of a sequence of presented screens as shown in Fig. 2B. The presented analyses in this work focused on data from task 3 (the deception trials). In this work, we define every CORRECT feedback as WIN condition, and the rest are LOSE conditions.

#### Data acquisition

EEG data were collected using DSI-24 system (Wearable Sensing, CA, USA) using 24 channels. Channel locations are P3, C3, F3, Fz, F4, C4, P4, Cz, A1, Fp1, Fp2, T3, T5, O1, O2, F7, F8, A2, T6 and T4 according to the international 10–20 system. The reference sensor is placed at the nominal Pz position, while the ground at the earlobes. The EEG data were digitized with 300 Hz sampling rate.

### Data analysis and feature extraction

A FIR band-pass filter with cut-off frequencies 0.1 and 40 Hz was used to filter the data. Then, three seconds of multichannel EEG data time-locked to the visual feedback (WRONG, CORRECT or TOO SLOW) presentation was extracted for each trial. For the baseline EEG data, the 2 min of rest-EEG were divided into three seconds non-overlapping windows of EEG, and each 3 s window is considered as one trial with a total number of 40 trials. Temporal and spectral features were calculated from the EEG signals collected from the frontal EEG channels; F3, Fz and F4. Welch’s periodogram method of power spectral estimation^53,54^ was used to calculate frequency-based features. A total of eight spectral and temporal features were calculated for each trial. The spectral EEG features were calculated as follows; for the frontal EEG channels F3 and F4 combined we calculated (1) the total power, (2) the absolute power difference between F3 and F4 in the frequency range (4–30 Hz). Moreover, for the EEG frontal channels, F3, Fz and F4, we calculated the following features, the total power in the (3) Theta (4–7 Hz), (4) Alpha (8- 15 Hz), and (5) Beta (16- 30 Hz) frequency bands for each channel separately.

Moreover, for the EEG frontal channels, F3, Fz and F4, we calculated the following temporal features; (6) the P300 which is a positive change in the EEG around 300 ms after the stimuli, (7) the FRN which is a negative deflection occurring between 200 and 300 ms and (8) the late positive potential (LPP), which is the average EEG calculated over a window (850–1600 ms) time-locked to the feedback presentation during this game. Finally, these features were normalized using z-score normalization and concatenated to form a feature vector.

### Feature selection and classification

**Figure 3:**
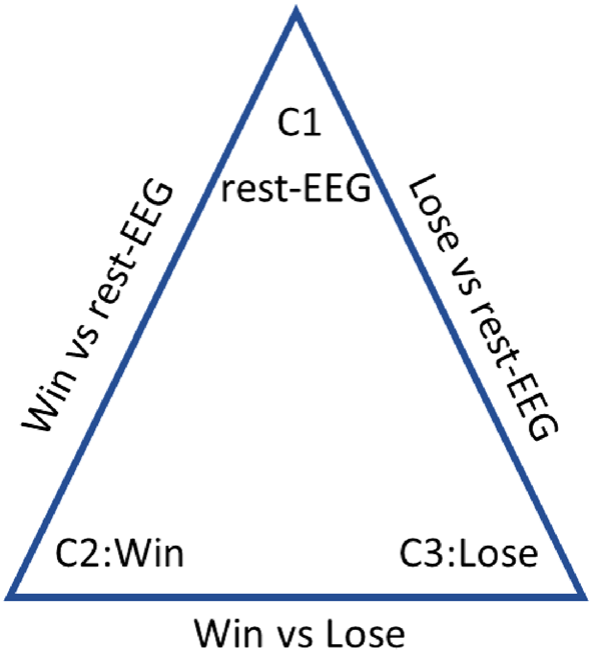
Diagram illustrates the three classification problems and the associated data classes collected and grouped from the EEG data collected during the third task of the proposed card game. The vertices of the triangle show the three classes, while the edges represent the three classification problems.

**Figure 4:**
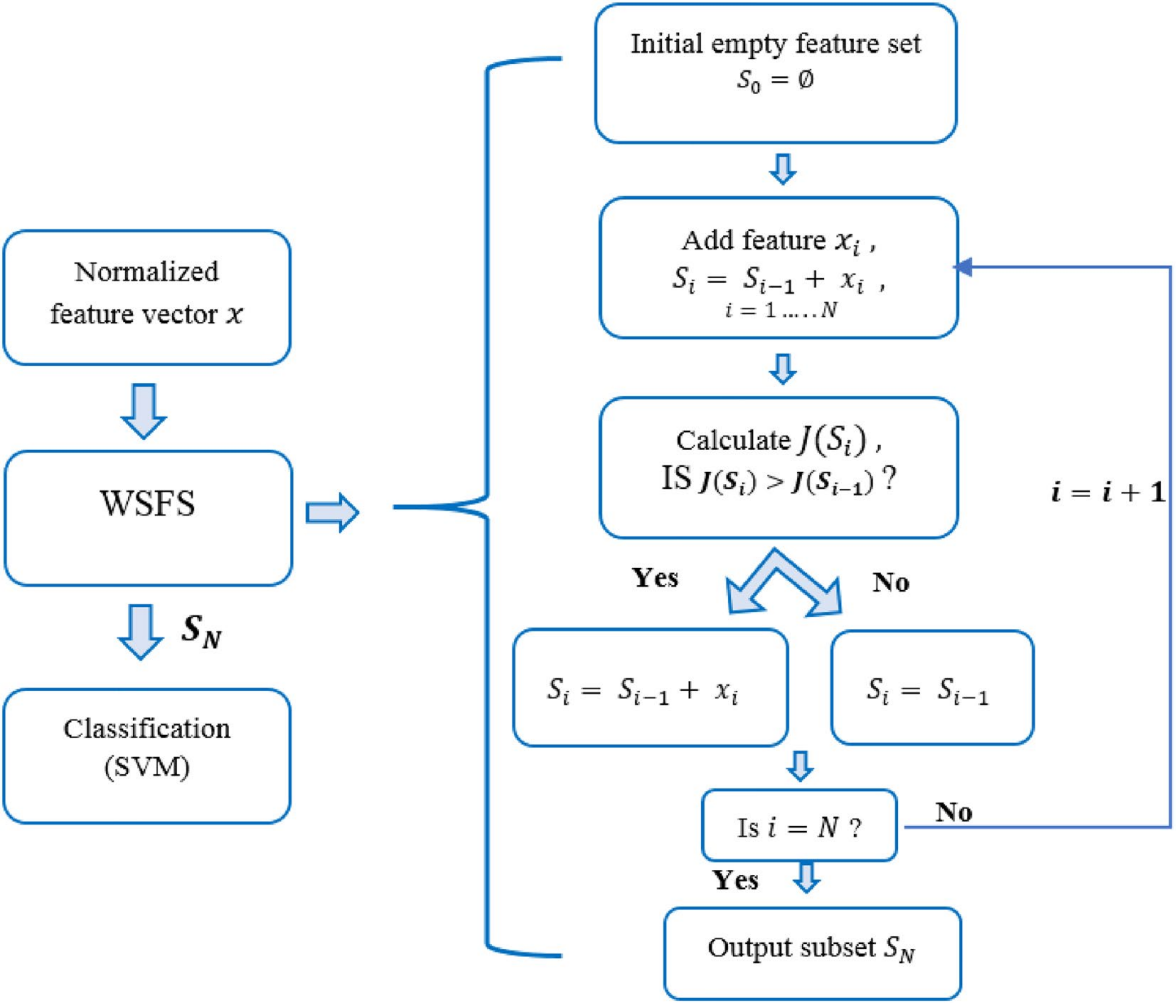
Classification steps and the weighted sequential forward selection algorithm.

A Support Vector Machine classifier (SVM) was used to evaluate the effectiveness of the extracted features in discriminating between three classes of data as shown in Fig. 3. Three (two-class) classifiers were designed using radial basis functions kernel. To avoid overfitting, a fivefold cross validation was used to train each classifier with a chance level of 50%. A sub-set of the extracted temporal and spectral features is selected based on the performance of each feature in discriminating between (1) distress and non-distress conditions represented by LOSE and WIN conditions, respectively; (2) LOSE and rest-EEG (baseline) conditions; and, (3) WIN and rest-EEG (baseline) conditions., as shown in Fig. 4. The weighted sequential forward algorithm was used to obtain most informative features that significantly contribute to the overall performance of each one of the three classifiers. Weighted sequential forward selection (WSFS) algorithm is a feature selection technique which has two main components, an objective function and a sequential forward selection algorithm. The selection algorithm uses bottom-up search starting from an empty set of features and gradually adding features which maximizes the classifier performance. For the objective function, we used the misclassification rate to minimize over all feasible feature subsets for classification and maximize the sensitivity of each class as shown in Fig. 4.

More specifically, given a feature ${\text{set}} = \{x_{i} { |}i = 1,2,3, \ldots N\}$ , where *N* is the total number of features. The selection algorithm forms an initial empty subset $S_{0}$ and gradually add each feature, $x_{i} { } \in { }x$, one at a time which maximizes the correct identification of both classes for each classification problem shown in Fig. 3. The cost function was chosen in a way to maximize classification rate of the two classes while maintaining a balance between their correct-classification rates, represented by their sensitivities. The cost function for the classification between the distress and non-distress conditions can be written as follows,

$$\begin{aligned} & J\left( S \right) = w_{distress} *Sensitivity_{distress} + w_{non - distress} *Sensitivity_{non - distress} , \\ & {\text{s.t}}{.}\quad w_{distress} + w_{non - distress} = 1 \\ \end{aligned}$$

The final selected subset of features after *N* iterations, $S_{N}$ where $size\left( {S_{N} } \right) \le size\left( x \right)$, can be written as:

$$S_{N} = \left\{ {x_{k} , \ldots x_{M} } \right\} = \arg_{M} J\{ x_{i} |i = 1,2, \ldots N\}$$

where $size\left( {S_{N} } \right) = M$ after $N$ iterations. For each participant, the WSFS algorithm generates three subsets of features {$S_{N1} , S_{N2}$ and $S_{N3}$} such that $S_{Ni}$ representing the most informative features contributing towards the accuracy of i^th^ classification problem, as can be seen in Fig. 4. For each participant, considering the features selected for $\{ S_{N1} , S_{N2}$ and $S_{N3}$} and recalling that we are considering a total of eight potential features, a score vector was then generated as follows,

$$Score_{p,q} = \left\{ {y_{1} ,y_{2} , \ldots .y_{8} } \right\}$$

where *p and q* are the participant and classification problem number, respectively, where ${\text{p}} = \left\{ {1,2, \ldots 21} \right\}$ and ${\text{q}} = \left\{ {1,2,{ }3} \right\}$. The score $y_{1}$ is the score of the first feature, which takes the value of one if this feature was selected by the WSFS for a specific p and q, and zero otherwise. After that, and for each classification problem, the score vectors are summed across participants and the percentage of each feature is plotted, as presented in Fig. 1.

The analysis introduced in this work is based on the EEG data collected during the third task. For each classification problem presented in Fig. 4, we calculated the accuracy and sensitivity of each class per participant. We also calculated the F1 scores. The accuracy reflects the ratio of the total number of correctly identified trials over the total number of trials for both classes. While the sensitivity of a class represents the proportion of the correctly identified trials of this class. F1^40^ score is also used to assess the performance of the three developed classifiers. This metric considers both the precision and recall of the test, precision is the ratio of correctly predicted patterns to the total predicted patterns; and recall is the ratio of accurately-recognized observations to the total actual observations^55^.This score results in value of 1, at perfect precision and recall values, and zero at worst.

### Validation methods

In order to validate the significance of the selected set of features in separating between (1) Win versus rest-EEG, (2) Lose versus rest-EEG and (3) Win versus Lose conditions, we performed one-sided Wilcoxon rank statistical test for each one of these classification problems. For each one of these problems, we selected three features which showed the highest percentage of selections by the WSFS algorithm. Then for each class, in each classification problem, we generated a feature vector containing the values of these selected three features for all the trials in this class. After that, we used the Wilcoxon rank statistical test to assess whether the difference between these two classes represented by these features, is significant or not. Therefore, for the Win versus rest-EEG, the total frontal power, FRN and P300 were selected. For the lose versus rest-EEG, the total frontal power, LPP and P300 were chosen, and finally for the Lose versus Win, the total frontal power, the absolute power difference of F3 and F4 and P300 were selected. The Wilcoxon rank test is a nonparametric hypothesis test, which returns the *p* value for the null hypothesis. The null hypothesis states that both feature vectors, representing both classes in each problem, come from the same distribution^56^. While, the alternative hypothesis states that both vectors are different, which means that the two classes are separable. Moreover, we applied the Wilcoxon rank test on the generated SVM scores from each classification problem. This was done to see whether these scores are significant to separate between each pair of classes for the three classification problems shown in Fig. 3.

Another test of validation was done to determine the significance of the selected set of features by randomly permuting the features and accordingly assigned randomly selected features to each participant assuring that each participant will have different subset of features than the features selected through feature selection algorithm. We repeated this permutation 100 times, and for each repetition we performed classification. Then, average performance measures (accuracy and F1 score) were calculated for Win versus Lose, Lose versus rest-EEG and Win versus rest-EEG classifications for each participant. Moreover, we used the right-side Wilcoxon rank test with significance level of 0.05, to compare the accuracy and F1 score values obtained through permutation with the accuracy and F1 score values obtained through classifications that used features selected based on feature selection algorithm.

## Conclusion

An EEG-based BCI system based on Affective Posner task was introduced. This system was designed to identify the changes in brain activity of individuals with ASD during rest, non-distress (WIN) and distress (LOSE) conditions. Furthermore, the analysis of the recorded EEG during the third task resulted in extracting EEG features that significantly contributed towards the classification between these conditions on a single trial basis. The P300 and total power in the (4–30 Hz) EEG frequency range, showed high accuracies in differentiating between distress and non-distress conditions. the P300, FRN and total frontal power contributed significantly in the WIN versus rest-EEG classification. In addition to these three EEG features, the LPP showed high contribution towards the classification of LOSE and rest-EEG conditions with low variance across participants. These results align with earlier findings about EEG features that can be extracted from neurotypical and individuals with ASD in response to emotional stimuli. This work is a first step towards building a real-time BCI system that could distinguish between non-distress (WIN) and distress (LOSE) on a trial by trial basis. Now that we have shown that EEG can reliably distinguish between distress and non-distress conditions), in future work, we aim to add capabilities to the presented BCI system such that it could monitor brain responses through EEG related to ER activity to work along with current clinical behavioral treatment methods, such as EASE, as a technological intervention tool. Also, this system could be integrated with virtual reality setup to simulate distressing activities in real-life settings, to provide opportunities to practice ER strategies and get real-time, automated feedback. Through such an integration, the proposed EEG-guided BCI technology could be used to complement clinical treatments that focus on ER to supplement in-person sessions with a therapist, decrease clinician time spent with each patient, or provide exposure to key components of treatments for those who do not have access to a therapist. Moreover, such a BCI could support all the existing technological approaches to monitor and analyze the brain responses during technology-driven interventions. As changes in the EEG features indicate high distressed conditions, visual cues that will enforce the participants to use ER strategies will be presented through the EEG-based BCI system during real-time ER intervention.

## Supplementary information

Supplementary information
