# Supplementary material for: Trial by trial EEG based BCI for distress versus non distress classification in individuals with ASD
Source: Sci Rep. 2021 Mar 16;11:6000. doi: 10.1038/s41598-021-85362-8 (PMC7971030; doi:10.1038/s41598-021-85362-8)
Supplement: Supplementary file 1 — Supplementary information [file 41598_2021_85362_MOESM1_ESM.docx]

**Trial by trial EEG based BCI for Distress vs Non distress Classification in Individuals with ASD – Supplementary Material**

Safaa Eldeeb^1^, Busra T. Susam^1,^ Murat Akcakaya^1^, Caitlin M. Conner^2^, Susan W. White^3^, Carla A. Mazefsky^2^

^1^ Swanson school of Engineering, University of Pittsburgh, Pittsburgh, PA, USA

^2^ School of Medicine, University of Pittsburgh, Pittsburgh, PA, USA

^3^University of Alabama, Tuscaloosa, AL, USA

The distribution of the selected features for each individual for each classification problem is shown in the supplementary figures S1-S3. The pie chart in each figure represent the percentage of the selected features for each classification problem for all the participants, and the tables show which features were significant for each participant. The value of 1 indicates that this feature was significant, while the value of 0 indicates otherwise.


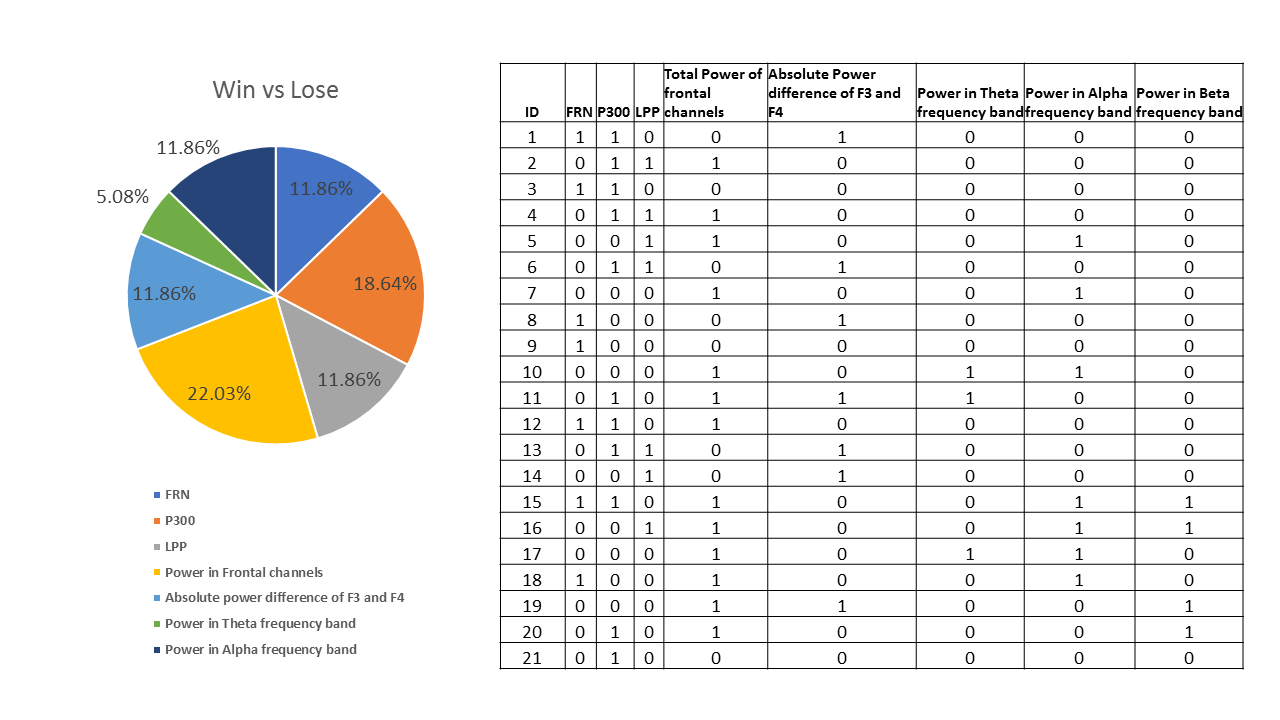
Supplementary figure S1. The most informative features of the classification of Win vs Lose.


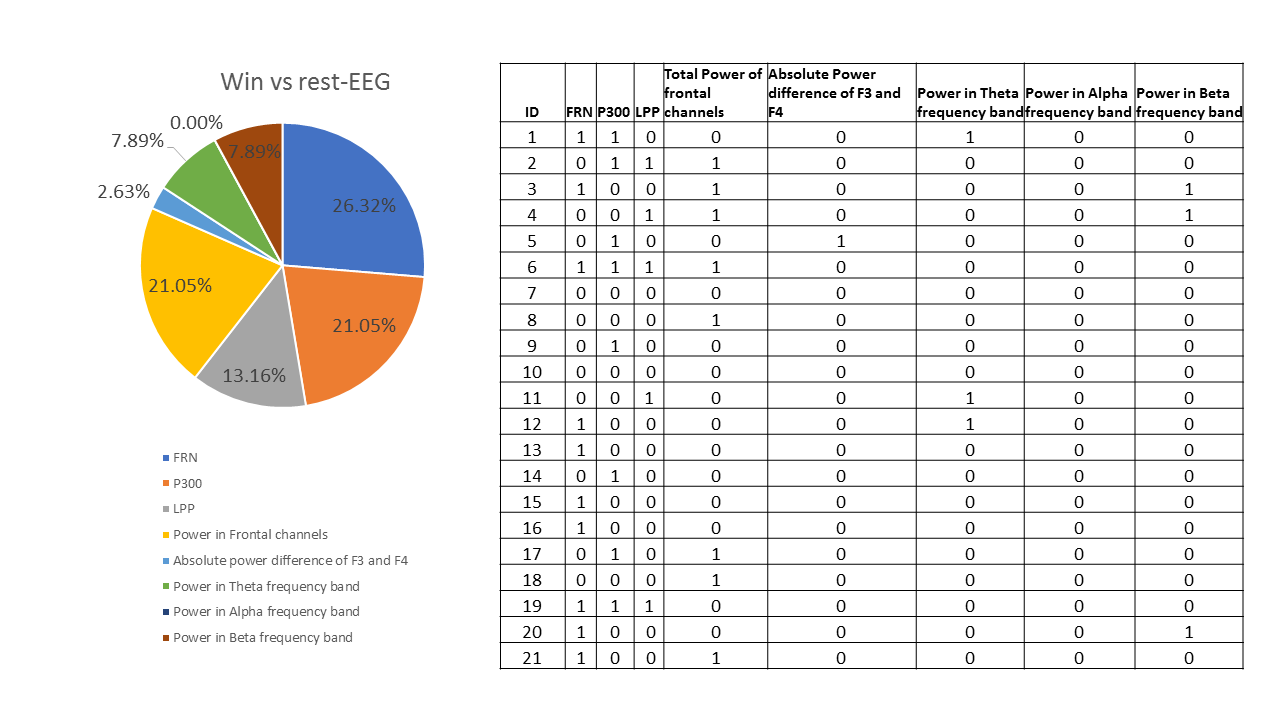
 Supplementary figure S2. T he most informative features of the classification of Win vs rest-EEG.


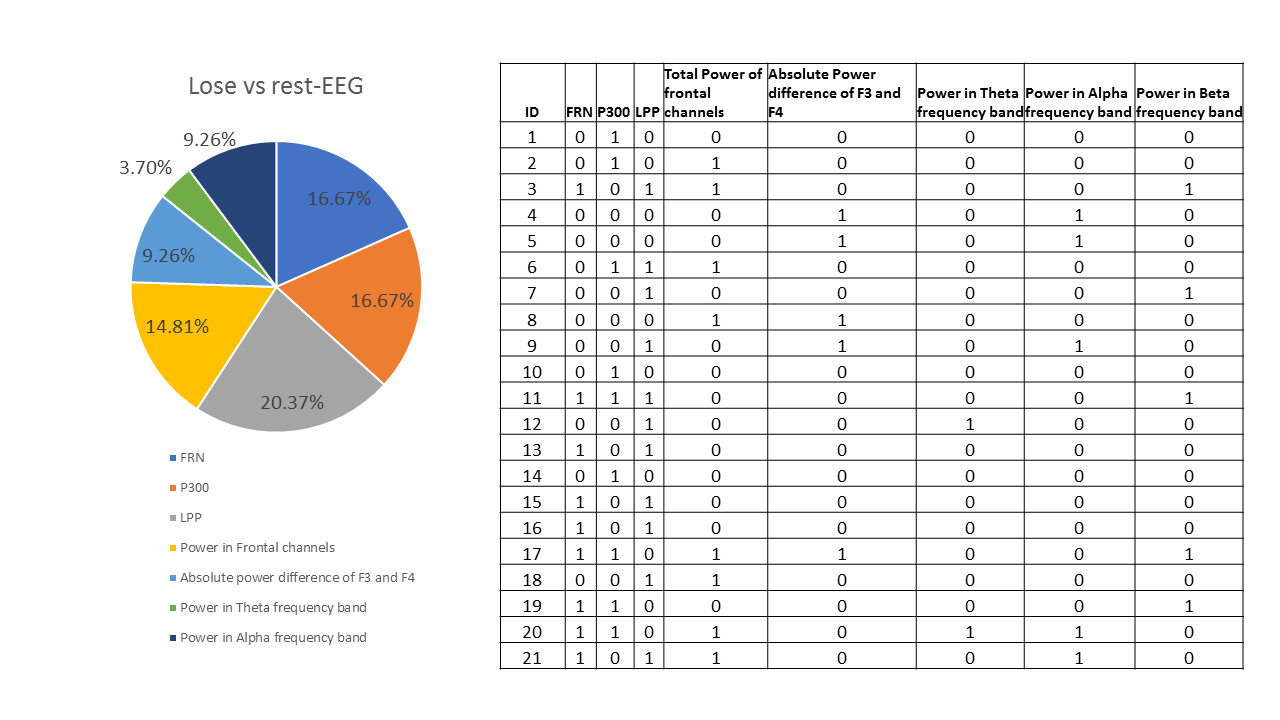
 Supplementary figure S3.The most informative features of the classification of Lose vs rest-EEG.
